# Secondary somatosensory cortex of primates: beyond body maps, toward conscious self-in-the-world maps

**DOI:** 10.1007/s00221-020-05727-9

**Published:** 2020-01-21

**Authors:** Rafael V. Bretas, Miki Taoka, Hiroaki Suzuki, Atsushi Iriki

**Affiliations:** 1 Laboratory for Symbolic Cognitive Development, RIKEN Center for Biosystems Dynamics Research, Kobe, Japan; 2 Graduate School of Social Informatics, Aoyama Gakuin University, Tokyo, Japan; 3 Azrieli Program in Brain, Mind and Consciousness, Canadian Institute of Advanced Research, Toronto, Canada

**Keywords:** Primate brain evolution, Projection, Holistic self, Self-consciousness

## Abstract

Recent human imaging studies have revealed the involvement of the secondary somatosensory cortex (SII) in processes that require high-level information integration, such as self-consciousness, social relations, whole body representation, and metaphorical extrapolations. These functions are far beyond its known role in the formation of body maps (even in their most complex forms), requiring the integration of different information modalities in addition to somatosensory information. However, no evidence of such complex processing seems to have been detected at the neuronal level in animal experiments, which would constitute a major discrepancy between human and non-human animals. This article scrutinizes this gap, introducing experimental evidence of human and non-human primates’ SII functions set in context with their evolutionary significance and mechanisms, functionally situating the human SII as a primate brain. Based on the presented data, a new concept of a somatocentric holistic self is proposed, represented as a more comprehensive body-in-the-world map in the primate SII, taking into account evolutionary aspects that characterize the human SII and its implication in the emergence of self-consciousness. Finally, the idea of projection is introduced from the viewpoint of cognitive science, providing a logical explanation to bridge this gap between observed behavior and neurophysiological data.

## Introduction

Where are we? Where is our body located? The answer may be self-evident to laypeople. For example, a person may describe oneself as being in an office, sitting stably on a chair in front of a computer monitor displaying this article. The description may continue, describing the person’s right hand holding the mouse, the right foot crossing over the left. The answer to these questions deliver two important messages to neuroscientists. One is that the body is assimilated in the world*.* The position of the body is perceived in reference to external objects, such as the monitor and the mouse in the surrounding environment. In other words, objects in the environment that exist independently of the individual are adopted as references when recognizing one's own body position in the frame of the external world—an antithesis of the commonly studied body-centered representation of the world—to produce bodily actions in the environment. The second important message is that the self and its physical body are recognized as a coherent whole*.* Although parts of the body, such as the trunk, arms, and legs, are manipulated separately to describe the bodily position, collectively, perhaps together with proprioception and even with visceral sensory inputs, they constitute the information of the body, which in turn produces a sense of the body as this coherent whole. This sense cannot merely be a somatotopic representation of the body (body maps) in the primary somatosensory cortex, but rather a mechanism beyond that, allowing the supervision and evaluation of these maps.

Located in the region of the parietal operculum, in the upper bank of the lateral sulcus adjacent to the primary somatosensory cortex, the secondary somatosensory cortex (SII) is known to respond to somatosensory (Robinson and Burton 1980) and visual (Hihara et al. 2015) stimuli. More recently, human functional brain imaging studies have expanded the potential functions of the secondary somatosensory cortex (SII) while investigating responses to touch, suggesting that this region may be a potential site where the aforementioned high-level information is processed [e.g., self-consciousness and self-location (Ionta et al. 2011), social relations (Blakemore et al. 2005), whole body image and self-recognition (Devue et al. 2007), and metaphoric extrapolations (Lacey et al. 2012)], requiring the integration of different modalities of distant, external information with proximal and intrinsic somatic sensory information. However, no evidence of such integrative processing has been detected at the neuronal level in animal experiments, which constitute a major discrepancy between humans and other animals, including non-human primates. This may happen due to either non-human primates having not evolved such capacities in the first place or due to non-human primates having those capacities but simply lacking the means (typically language in humans) to report phenotypes of high-level information processing to the experimenter.

Alternatively, these mechanisms and phenotypes may exist both in human and non-human animals (although at different degrees), but researchers have not yet developed theories that could unfold the principles of sense of the body information processing and phenomena. The current rising notion of projection from the field of cognitive science might provide clues to understanding this sense and help avoid falling on an infinite regress fallacy of increasingly complex supervising layers. The notion of projection, in short, is an ideal mental function that maps (i.e., projects) contents of the internal model of the self and the perceived world onto actual physical environmental worlds (“The notion of projection”). However, its neural underpinning is yet completely unknown, and the mechanisms of SII information processing might provide a clue about the neural correlates of projection.

### The notion of projection

Few researchers in the cognitive neuroscience community doubt that people receive information from objects and events in the outside world and construct their neural representations accordingly. Based on this assumption, cognitive neuroscientists have been studying how neural representations are generated by specifying the activated regions, the route of information flow, and resultant brain networks. This type of research can be called a reception-construction one.

Reception-construction research, however, is only one side of the coin, since neural representations are in most cases localized in the real world. For example, in vision, the reflection of light from a particular object is received in the retina. It passes through the optic nerve and stimulates various regions in the brain, which results in the construction of the neural representation of the object. However, this is not the end of the story, because the object is not perceived in the brain, but rather in a specific location in the world (Pylyshyn 2011). Certainly, there are neural mechanisms that code positions of the world, such as grid cells and place cells (Hafting et al. 2005; O’Keefe and Nadel 1978). However, it does not mean that the object appears in the entorhinal cortex or hippocampus. The same can be said about the somatosensory sensation discussed in this paper. When something touches the hand, a specific part of the primary somatosensory cortex becomes active. However, the feeling at that moment occurs in the specific part of the hand where the stimulus was given, not in the primary somatosensory cortex.

What has been mentioned so far strongly suggests the need for the mechanism of projection that maps internally constructed neural representation to the world including one’s own body. Without this projection, internal representations would hang in the air. The same argument was made almost a half century ago by Michael Polanyi in his seminal book The Tacit Dimension (Polanyi 1967). He took the example of a blind man's stick. When the stick hits something, he will feel its impact against his fingers and palm. But, at the same time, the awareness of its impact on his hand is transformed into a sense of its point touching the object. He referred to feelings of the hand as proximal terms and external object that causes the bodily feeling as distal ones. He then pointed out that only through projecting from the former to the latter would a comprehensive understanding be made possible. If his proposal were to be translated into a more modern context, the proximal term would be regarded as the neural representation, since the sensation of the body is created by the state of the brain.

Projection is too obvious for humans because, in many cases, we carry out the projection automatically and without any difficulties. This makes it difficult to explore its mechanisms and processes. However, using various experimental techniques, projection can be controlled or distorted. As vision science has achieved dramatic developments by using illusions, the research on projection could follow suit by using such unusual projections. For example, in the rubber hand illusion, somatosensory activity is projected not on the real hand, but on the fake hand by unusual visuo-tactile synchrony (Botvinick and Cohen 1998). Also, as seen in the ventriloquist effect (Alais and Burr 2004), the source of the sound is mistakenly identified, due to the unusual interaction between auditory and visual information.

The use of tools also modifies and distorts projection. As seen in the cane of the blind, the feeling of the palm produced by the obstacle is projected onto the obstacle at the tip of the cane rather than at the palm of the hand. The use of VR/AR also changes the projection. In playing well-designed VR games, people project their bodies not onto the real world, but in the imaginary one (Bailenson 2018). Even if there is no clear stimulus source in the outside world, people sometimes project an internal representation formed in the brain into the real world, e.g., patients with schizophrenia experience hallucinations in the absence of external stimulus sources (Frith et al. 1996). In addition, under extreme circumstances where abnormalities occur in our senses, persons that should not exist are sometimes perceived (Geiger 2009).

Since many of the irregular projections mentioned above are caused by discrepancies between information obtained from sensory organs that should originally co-occur in a harmonious way, multi-sensory integration should be the key mechanism of projection. Certainly, one component of the projection must be somatosensory information stored in the primary somatosensory cortex (SI). However, neural representations at SI do not code relational information linking the body and the world. Thus, to make the projection possible and create the body-in-the-world representation, neural representation at SI must be incorporated with other representations that code information about the external world. Candidate representations to be incorporated are visual, auditory, and vestibular ones that code environmental information as well as relations between the self and environment. SII is one of the regions that receives inputs from all of these representations. More importantly, we found clear evidence that neurons in SII respond not only to tactile information, but also to visual and vestibular information.

In this article, we try to integrate theoretical and empirical frameworks in a complementary manner, proposing a novel concept on the function of the primate secondary somatosensory cortex, which has neither been sufficiently studied nor fully understood until now. In this attempt, we first overview the current status of experimental evidence on human and non-human primates’ SIIs to expound the discrepancy between current experimental results (“Somatotopic functions of SII and adjacent opercular cortex: a conventional view” and “Multisensory integration”). Following that, recent neurophysiological reports with potential to fill this gap are introduced (“Recent advances in SII neuroscience: evidence from primates”). Based on these and together with recent findings in molecular genetics (“What is special about the primate parietal operculum? Preadaptation for human SII”), further considerations are made in relation to the evolutionary aspects and the mechanisms of SII that could explain human SII functions as a logical development of the primate brain. Finally, we propose the new concept of a somatocentric holistic self represented as a body-in-the-world map in the primate SII (“Expansion of the information space: transition from body map to body-in-the-world map” and “Conclusion: conscious experience of the self in the world”). According to this concept, the self is formed through a two-way interaction between body and environment, relativizing the body limits by their perceived influence in the environment observed in the abnormal projections described earlier, and referencing the self by the reach of the body. These fluid limits characterize human SII high-level functions, eventually resulting in implications to the emergence of self-consciousness through the conceptual framework of projection from the cognitive science.

## Somatotopic functions of SII and adjacent opercular cortex: a conventional view

### Multiple body maps

**Fig. 1:**
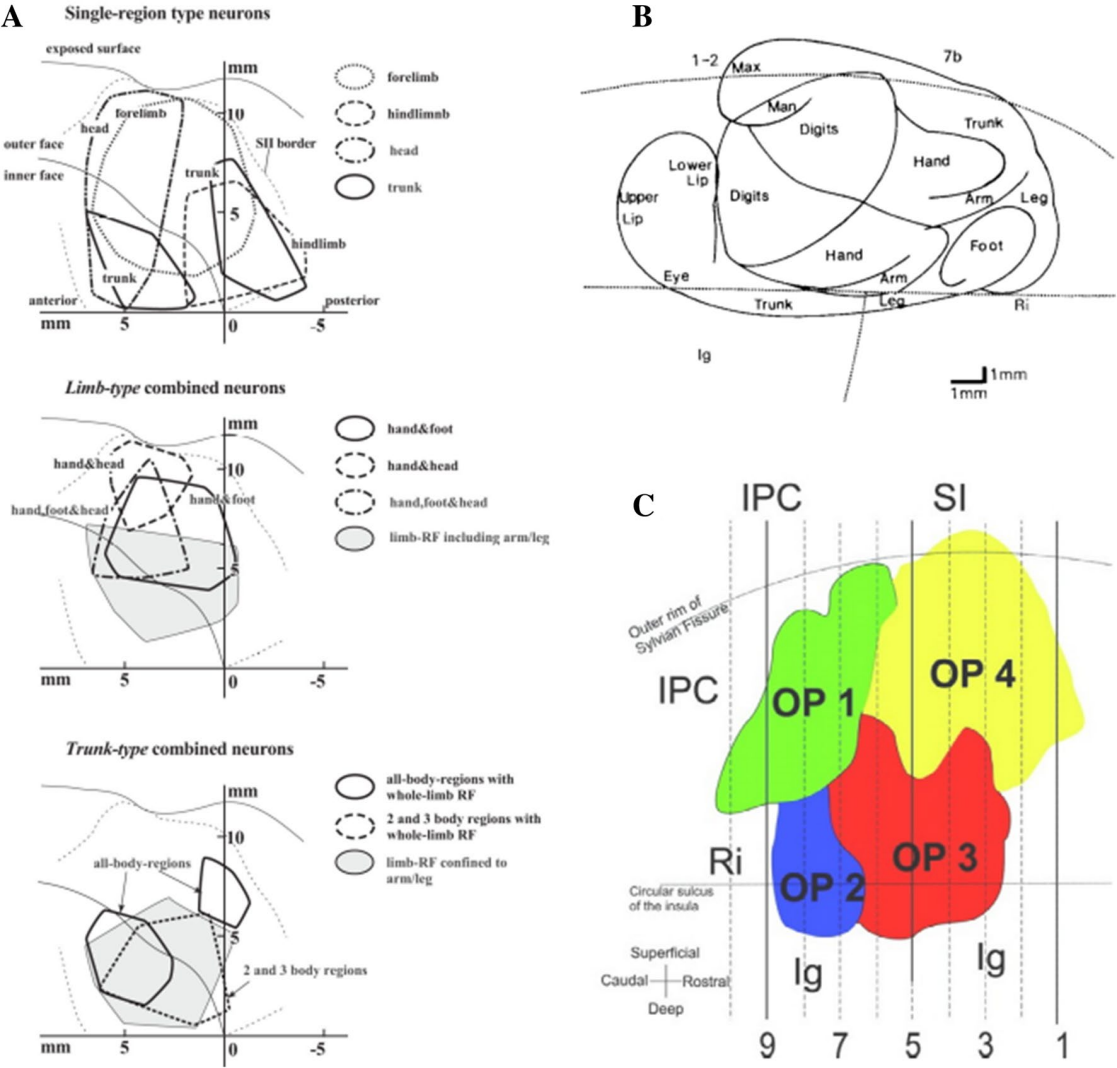
Cortical organization of the parietal operculum. **a** Unfolded surface of the lateral sulcus of macaque showing the mirrored distribution of somatosensory responsive neurons of different body parts (electrophysiological data). The vertical axis indicates distance from the upper fundus (Taoka et al. 2016). **b** Distribution in the SII of anterograde tracers injected into closely related cutaneous responsive sites in macaque (Burton et al. 1995). Same representation format as (**a**). **c** Flattened representation of the four cytoarchitectonic areas in the human parietal operculum (OP for operculum). Indicated on figure: inferior parietal cortex (IPC), retroinsula (Ri), primary somatosensory cortex (SI), and granular insular cortex (Ig). OP 4 and OP 1 are suggested to be the human analogues of the primate parietal ventral area (PV) and SII, respectively (Eickhoff et al. 2006)

Lateral to the primary somatotopic representation in the postcentral somatosensory cortex (SI), the upper bank of the lateral sulcus of the primate brain was believed to possess an additional body map (Robinson and Burton 1980). Through electrophysiological studies using anesthetized macaques, Krubitzer et al. (1995) showed the existence of two complete somatotopic maps aligned in mirror images—the secondary somatosensory area (SII) and the parietal ventral area (PV), seen in Fig. 1a. Robinson and Burton (1980), in a neural connection study, unveiled two somatotopic maps in SII when retrograde tracers were injected in previously known body regions in area 3b and area 1 of the primary somatosensory cortex (Fig. 1b). Eickhoff et al. (2006) found that the human parietal operculum could be divided into four areas (OP1–OP4; Fig. 1c) by a quantitative cytoarchitectonic analysis using postmortem brains, in which OP1 and OP4 correspond to the two areas, SII and PV, defined by Krubitzer et al. (1995). As outlined above, there is a controversy in defining two separated somatosensory regions within the previously called SII area. While some studies say there are two somatosensory areas (PV and SII), others claim that the two areas exist within the SII; therefore, in this review we consider the whole somatosensory area in the upper bank of the lateral sulcus as the secondary somatosensory area (SII) that includes both PV and SII (OP4 and OP1), as defined by Krubitzer et al. (1995).

### Tactile perception

Numerous unit-recording studies using awake macaque monkeys have been carried out to elucidate the functions of the SII. These studies often showed complex neural responses, such as attentional modulation of neural responses and large somatic receptive fields (RFs) covering more than one body part as well as both sides of the body. Although these results suggest that SII is engaged in more complex information processing than SI, its functional roles (besides the integration of the tactile information needed for object recognition) remain to be clarified. Hsiao (2008) investigated response properties of hand neurons in SII to precisely controlled stimuli, revealing that their RFs vary in size and shape, consisting of orientation-tuned fingerpads among untuned excitatory or inhibitory fingerpads, which suggests that these RFs are the kernels underlying tactile object recognition.

Romo and de Lafuente (2013) analyzed SII neural responses in monkeys performing a frequency discrimination task. The researchers then compared the neural responses with those in other cortical areas, such as SI, premotor, and prefrontal areas. They showed that SII neurons do not represent the temporal structure of the stimulus, but they revealed the presence of two different frequency-dependent populations (with positive and negative monotonic functions), contributing to stimulus discrimination. Given SII’s neuroanatomical connections, converging information from SI, and premotor and prefrontal cortices (Cipolloni and Pandya 1999; Disbrow et al. 2003), SII would be the region where sensory data is translated into perception.

In humans, skin palpation activates the contralateral SI and bilateral parietal operculum including SII, but regions that contribute particularly to tactile perception are undetermined. Preusser et al. (2015) showed, by voxel-based lesion-symptom mapping, that in brain-damaged patients with an intact SI but impaired touch perception, the symptoms were related only to a single cluster across the contralateral parietal operculum, insular cortex, putamen, and the underlying white matter towards prefrontal structures, indicating that these brain structures, including SII, are supposedly responsible for the perception of touch.

### Sensorimotor integration for object manipulation and active tactile discrimination

In human brain imaging studies, SII activation during motor execution may have a role in sensorimotor integration. Binkofski et al. (1999) demonstrated the activation of SII among other cortical areas (ventral premotor cortex, anterior intraparietal sulcus, superior parietal lobule) during manipulation of meaningless complex objects (thus, sensation without perception), suggesting the involvement of these areas in object manipulation. Hinkley et al. (2007) found increased bilateral activation of SII/PV and their rostrally adjacent region (PR) in a tactile task, with subject’s movement compared to a tactile-only condition, indicating this area may play a role in the somatomotor integration necessary for manual exploration and object discrimination. Valenza et al. (2001) reported a case of tactile apraxia (i.e., a patient with severe impairment only in active tactile object exploration but with intact passive tactile recognition), suggesting that the patient lacked a specific ability in using tactile feedback to generate the necessary exploratory procedures for tactile shape recognition. A functional MRI during sensory stimulation detected the preserved activation of SI, but lacked the activation of SII observed in the control subjects. Maule et al. (2015) showed that applying conditioning transcranial magnetic stimulation (TMS) over the parietal operculum (SII) modulated the output of the motor cortex when the information on object size was acquired haptically but not visually, suggesting that SII contains a haptic memory of objects’ macrogeometry and the appropriate motor plan for grasping them.

SII functions on sensorimotor integration using macaque monkeys have been rarely attempted. Fitzgerald et al. (2004) observed two proprioception-dominant hand areas in SII where neurons responded when a macaque actively grasped objects. Taoka et al. (2013) found SII neurons located around the region in between hand and face representations that became active during both eating and retrieving with the hand. Those neurons rarely responded to touching objects, but were associated with specific purposeful movements, such as picking up food, indicating that these neurons are involved in the monkey’s ability to recognize and understand events during the feeding behavior.

## Non-somatotopic processing: current view of human SII

In spite of SII somatotopy including neurons with large RFs covering more than one body part and both sides of the body, the above-mentioned functional neurophysiological studies using awake monkeys focused solely on tactile perception by hands or sensorimotor integration during object manipulation with hands or mouth. This leaves unexplored the potential SII functions of neurons with large and bilateral RFs, in which information from other sensory modalities could be potentially integrated (Robinson and Burton 1980), though such information has been generally believed to not exist. Despite this, recent human brain imaging studies have demonstrated SII activation by other sensory modalities, such as vision, audition, and vestibular stimulation. Furthermore, those studies have also proposed novel concepts of SII’s specific higher-order functions typical of humans.

### Multisensory integration

#### Visual activation

Several human fMRI studies have reported that SII is activated both when the subjects are touched and when they observe others being touched by objects, suggesting that SII neural mechanisms for one's own tactile perception also contribute to the understanding of others’ [see review by Keysers et al. (2010)]. Indeed, in some people with mirror-touch synesthesia (a symptom evoking unusual sensations of touch when observing others being touched), Holle et al. (2013) found a correlation between SII activity and subjective intensity measures of mirror-touch synesthesia and increased gray matter volumes within the SII of the synesthetes’ brains. In addition, the synaesthetes showed hypo-activity in posterior SII when watching a dummy (unlike other agents) being touched. Despite observation of others being touched commonly activating SII, as described above, normal subjects do not perceive being touched themselves. Thus, SII may be involved in self–other recognition processes, and relative enhancements of self-awareness caused by unusual activity of SII might result in mirror-touch synesthesia. This differentiation could be context dependent at the neuronal level, such as observed in the parietal cortex of monkeys during social interaction (Ishida et al. 2010). Disbrow et al. (2003) suggests that the SII is part of a network involved in acquisition, exploration and identification of objects, contributing to visually guided and movement and spatial recognition of the self and other bodies (Hihara et al. 2015). Future electrophysiological studies may be able to identify the nature of self/other recognition and localization in the SII.

#### Vestibular activation and influence

The vestibular cortex (parieto-insular vestibular cortex of monkeys or OP2 of humans; see Fig. 1) resides adjacent to SII, but has not been considered to overlap with SII. However, caloric vestibular stimulation (CVS) to both healthy subjects and hemianesthesia patients clearly activates these opercular cortical areas including SII [see reviews by Bottini and Gandola (2015) and Ferrè and Haggard (2015)]. A PET study by Bottini et al. (1995) found that those areas activated by CVS overlapped areas activated by the tactile stimulation of the contralateral hand, involving the SII together with the putamen, insula, premotor cortex, and inferior parietal cortex. Additionally, the modulation of somatosensory-evoked potentials by CVS has been reported in healthy human subjects. Ferrè et al. (2012) revealed that CVS specifically enhanced the N80 component [found to originate from OP1/SII (Eickhoff et al. 2010)] by median nerve stimulation in healthy humans, ruling out indirect effects by general arousal or supramodal attention.

Studies on healthy humans and brain-damaged patients showed the vestibular–somatic interaction enhancing somatic sensation through vestibular stimulation [e.g., a decrease in the tactile discrimination threshold in healthy humans can accelerate the recovery of tactile impairments or hemineglect symptoms (Ferrè and Haggard 2015)]. Caloric vestibular stimulation was also shown to alter the perceived size of the subject's own hand (Lopez et al. 2012). The fact that somatic modulation by vestibular stimulation is specific to SII suggests a possible role of SII in vestibular–somatic interaction. This role could involve integrating gravitational reference frames from the vestibular as well as pressure from the somatosensory system and/or encoding the position of the body or its parts in space (Lopez et al. 2008).

### Higher-order functions

#### Metaphorical processing

Lacey et al. (2012) showed in human fMRI that the texture-selective somatosensory cortex in the parietal operculum (i.e., OP1 that occupies the posterior part of SII) is activated when processing sentences containing textural metaphors in comparison to literal sentences matched for meaning, suggesting that texture discrimination in SII is processed at the level of metaphorical perception. This supports the idea that the comprehension of metaphors is perceptually grounded (i.e., structured around metaphorical mappings derived from actual physical experiences of modality-specific information in SII, where the signal transmitted from SI is converted to information), contributing to perceptual decision making (Romo and de Lafuente 2013).

#### Social perception with empathy

SII-vicarious activation by the observation of someone being touched may contribute to the understanding of others’ somatosensory states, thereby contributing to social perception (Keysers et al. 2010). Indeed, impairments in affective empathy have been observed in patients with acute ischaemic temporo-insular stroke (Leigh et al. 2013). Furthermore, Blakemore et al. (2005) proposed the idea of mirror neurons for touch, underlying empathy toward others’ somatosensory feelings, and SII is thought to comprise a part of this neural mechanism. Corroborating that notion, Nummenmaa et al. (2014) asked participants to watch movies of boxing matches either passively or while simulating a prespecified boxer’s feelings. When the subjects were asked to simulate the boxer’s feeling, brain activity increased in SI and SII cortices together with premotor, posterior parietal, superior temporal cortices, and the dorsal attention circuits. Thus, sharing a third person’s feelings synchronizes the observer’s own brain mechanisms of somatosensory perception, eventually supporting mutual social understanding and interaction. Interestingly, this empathy mechanism observed during touch may not be present for pain (Singer et al. 2004).

#### Self-body awareness

Hogendoorn et al. (2015) found that the P100 component of somatosensory-evoked potentials (considered to reflect SII activity and to indicate conscious perception of a tactile stimulus) is enhanced when the subjects touched their own symmetrically opposed limbs. This suggests SII’s involvement in ones’ awareness about his or her own body structures. Furthermore, when Hashimoto and Iriki (2013) presented healthy participants with original and distorted images of their own, whole bodies (but without head/face) and asked the participants whether the images were of themselves or not, perception of own body size and proportion was associated with bilateral inferior parietal activity including SII, suggesting a role related to awareness of the bodily self as a whole.

As for awareness of body parts, Corradi-Dell’Acqua et al. (2009) presented pictures of arms rotated at different angles while giving varied instructions to their participants. It was found that a part of the parietal operculum corresponding to OP1 (See Fig. 1c) was activated when the subjects were asked to imagine rotating their own arms until matching the orientation of the presented picture. The researchers concluded that SII could contain the neural substrate for the awareness of the body schema that codes the orientation of one’s body parts in space. In addition, sensorimotor deficits caused by lesions in the SII and surrounding areas are often accompanied by self-awareness impairments such as anosognosia and somatoparaphrenia (Karnath et al. 2005; Baier and Karnath 2008).

Tsakiris et al. (2007), using the rubber hand illusion, detected positive correlations between the strength of the sense of body ownership and activity in the right posterior insula and frontal operculum, and also a negative correlation with SII activity (perhaps representing a mismatch/conflict between tactile and visual information). They concluded that those structures would collectively link current sensory stimuli to one’s own body, therefore being involved also in self-consciousness. In addition, Brozzoli et al. (2012) found increased SII activity as well as an activation of premotor and posterior parietal cortices when visual stimuli were applied to the peri-rubber-hand space under the rubber hand illusion condition, suggesting SII’s involvement in the body ownership system.

## Recent advances in SII neuroscience: evidence from primates

As illustrated in the previous section, high-level information processing is assumed to happen in the human secondary somatosensory cortex, even though evidence at the neural level has been lacking in animal experimentations. In this section, in pursuance of bridging this gap, recent findings from our laboratory in the study of the SII of macaque monkeys, which seems to be a good candidate for representing the precursory mechanism of human SII, are discussed.

### Multimodal integrations

The largest mystery and discrepancy between the human and non-human SIIs is the existence of visual inputs, a mandatory requisite to integrate higher-order information processing. Complex (especially active) visual stimuli needed to be examined in consciously behaving monkeys. However, it has been generally believed that the monkey SII is devoid of visual input (Robinson and Burton 1980), except for indirect evidence of 2-deoxyglucose consumption (an indicator of neural activations) after viewing actions of others (Raos et al. 2014), and an fMRI study (Guipponi et al. 2015). These perspectives have been somewhat lacking in the conventional experimental design, which has been insufficient for exploring visual information in connection to body maps.

Hihara et al. (2015) exhibited rather complex visual stimuli to 1157 SII neurons (from eight hemispheres of six monkeys), detecting 306 responding neurons. Such visual stimuli contained natural and meaningful information, in contrast to previously shown simple visual cues (dark/light transitions, directed motion in the visual field), which were incapable of evoking responses in the earlier studies. These visual neurons were distributed continuously along the lateral sulcus covering the entire SII, along with other somatosensory neurons. Occasionally applying auditory stimuli to visual neurons also allowed the detection of ten auditory-responsive neurons, in addition to somatosensory responses. Hence, the structure of space that this information represents is not necessarily linked with the tridimensional physical space in which straightforward body maps reside, but a rather more complex and abstract one in which mathematical coordinates transformations/projections should take place. In this sense, this might be somewhat equivalent to frequency space (of tactile vibration), which is free from spatial body maps but transformed into other definitive structures (Romo and de Lafuente 2013).

### Whole body somatic sensory integration

A conventional body map is a topographical organization of body parts based on RF locations along the physical bodily structures. However, SII visual response properties, as depicted above, only vaguely correspond to multiple and large parts of the body structures. Then, how could tactile response properties relate to visual responses? Taoka et al. (2016) examined RFs of SII neurons under this perspective (i.e., how the RFs of SII neurons spread across four major body regions: head, trunk, forelimb, and hindlimb). About 25% of the RFs of recorded neurons (total of 1099 from nine hemispheres of six monkeys) covered combined body regions, and among those, 90% had RFs on bilateral body parts. Two tendencies of RF convergence were observed: (1) the distal parts of the limbs (i.e., hand and foot) and the mouth are interconnected, and (2) the trunk RFs extend continuously toward the distal parts of the limb and head to cover the entire body surface. These neurons may be viewed as aligned along the physical bodily structures, but their spatial resolution is too low to justify its usefulness. Moreover, the function of receptive fields covering the whole body cannot be the representation of the bodily spatial structure itself. Alternatively, it would be reasonable to assume there is another class of maps of the whole body, in which the body’s dimensions are detached from physical structures. This kind of information integration is supported by an anatomical demonstration of various cortico-cortical inputs converging onto the SII in monkeys (Taoka and Toda 2004). In addition to dense connections to SI, the cortico-cortical inputs include (1) the lateral bank of the intraparietal sulcus, (2) the anterior part of the ventral premotor area, (3) the cingulate motor area, (4) the inferior bank of the principal sulcus, and (5) the caudal part of the insular cortex.

### Body schema and tool use induced morphological plasticity

**Fig. 2:**
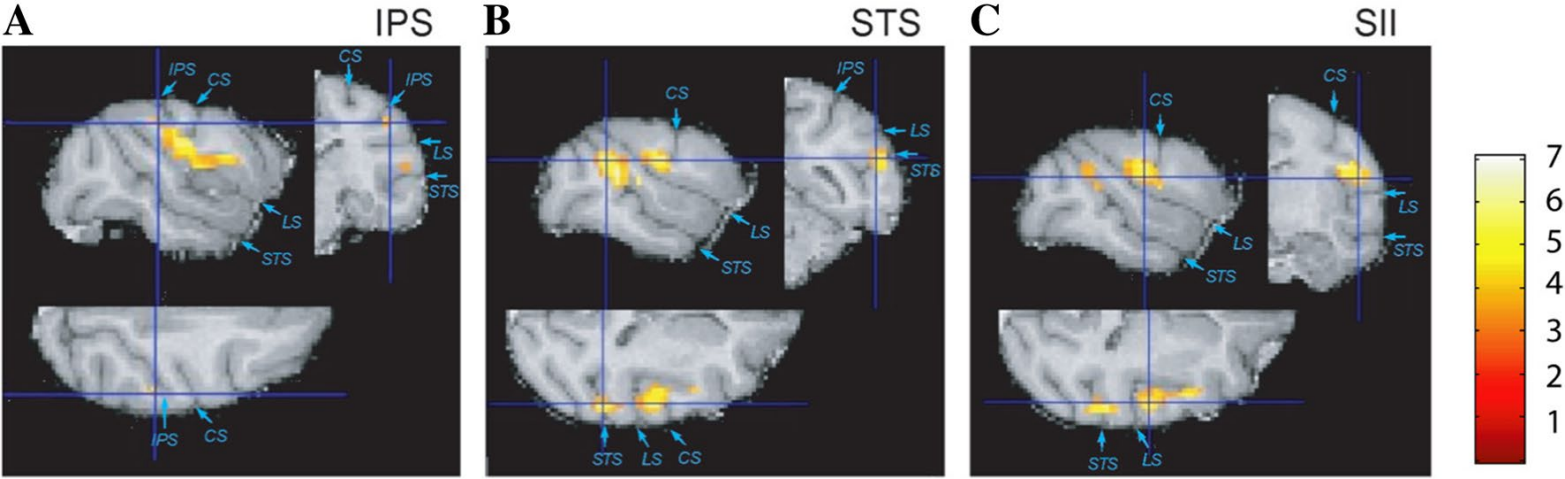
Gray matter increase in the right intraparietal sulcus (**a**), superior temporal sulcus (**b**), and SII (**c**) of macaque after training to use a rake to retrieve food that could not be reached otherwise. From (Quallo et al. 2009)

One recent, unexpected finding about monkey SII (yet relevant to the discussion on bridging the human–nonhuman gap) is the morphological plasticity of monkey SII in relation to tool-use learning. MRIs and voxel-based morphometry (VBM) were performed to detect changes in the brain structure of three adult Japanese macaques trained to use a rake (Quallo et al. 2009), revealing a significant increase in gray matter in correlation with rake performance. The effects were most significant in the superior temporal sulcus, SII, and intraparietal sulcus (Fig. 2). Gray matter volume in peak voxels increased by up to 17% during the intensive training period, while the earliest changes were seen after just 1 week of intensive training, generally peaking when performance on the task plateaued. This experiment was first aimed at detecting changes in the intraparietal sulcus, where neurons’ RFs adapt to code the tool as an extension of body parts; thus, SII expansion was unexpected. Despite this, the result is reasonable when assuming that the coding and manipulation of images and schemas of body parts (Corradi-Dell’Acqua et al. 2009) is the crucial principle for tool usage, and such a body image (accounting for the tool) is formed through the integration of somatosensory and visual information (Taoka et al. 2016), being possibly related to self-awareness and consciousness (Tsakiris et al. 2007). On the other hand, this finding also implies that information processing in SII is capable of plastic and dynamical changes, depending on situations and environmental requirements, rather than a precise and fixed mode depending on intrinsic bodily structures. This plastic nature is further corroborated by the localization of plasticity-related gene expression corresponding to the tool-use learning processes. Through a comprehensive analysis of genes related to synapse formation and function (ADAM19, SPON2, and WIF1), statistically significant differences in expression levels in neurons and glial cells were found (Matsunaga et al. 2015d).

## What is special about the primate parietal operculum? Preadaptation for human SII

**Fig. 3:**
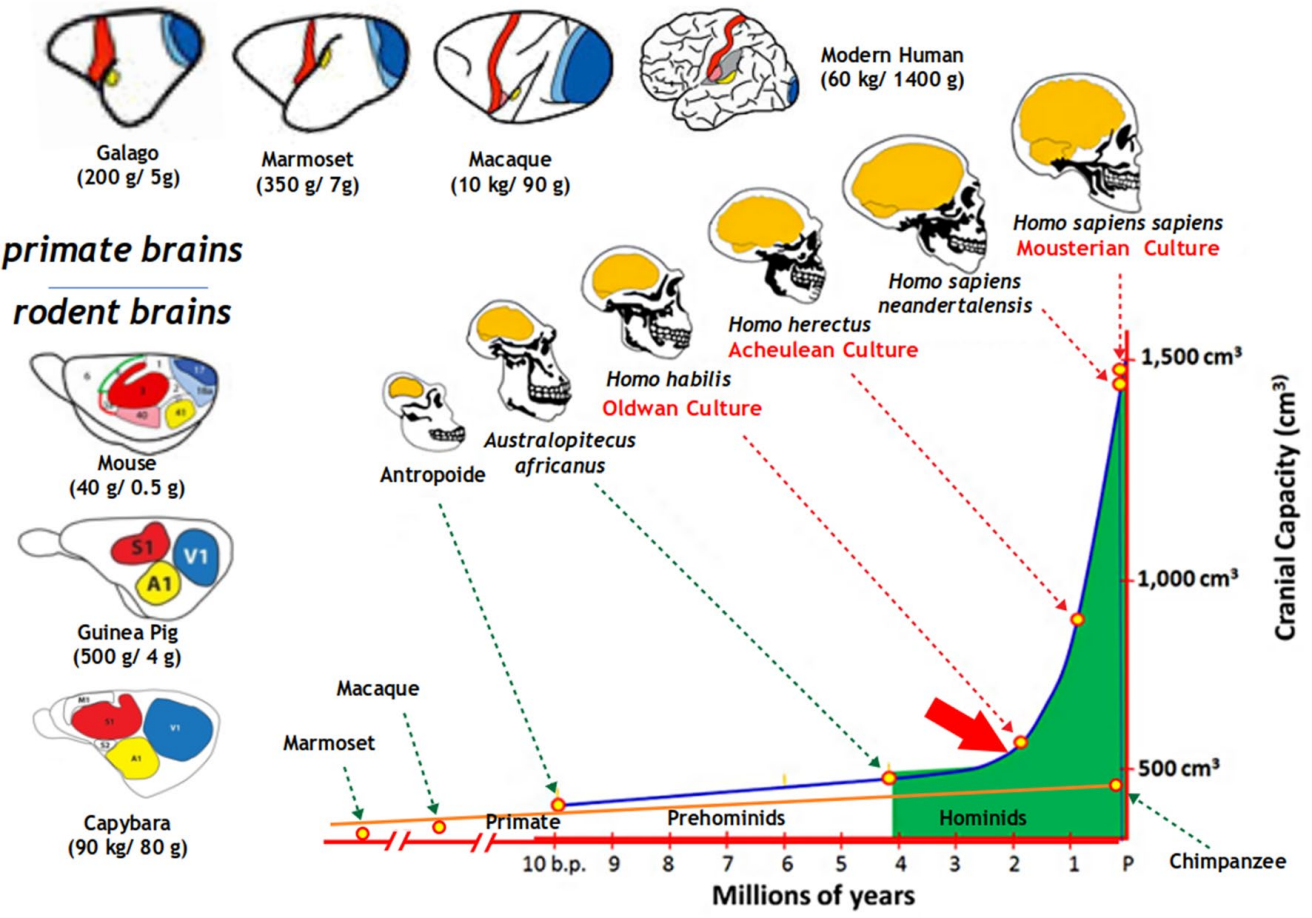
Transition of brain capacity (ordinate) along the evolutionary processes of various ancestral pre-hominids and hominids (abscissa) [adapted from “Evolution Du Volume Cérébral Des Hominidés” (2016)]. Inset illustrations of skull and brain depict representative hominids plotted on the graph. Brain expansion suddenly accelerated when *Homo habilis* started to use stone tools (oblique red arrow), and it branched off from the regression line (orange solid line) for extant non-human primates including great apes (ca. Chimpanzees). *Oldwan culture*: the earliest widespread stone tools were simple, usually made with one or a few flakes chipped off with another stone, and used by *Homo habilis*. *Acheulean culture*: stone tool characterized by distinctive oval and pear-shaped “hand-axes” manufactured and used by *Homo erectus*. Simple syntax of vocal communication, a primitive form of human language, is thought to be required for the transmission of this culture. *Mousterian culture*: techno-complex and symbolic archaeological industry of fling lithic technologies associated with *Homo sapiens neanderthalensis* throughout early *Homo sapiens sapiens*. Insets along top-left edges are diagrams illustrating different principles of brain organization between rodents (left; middle to bottom) and primates (top; left to right) [adapted from Dooley and Krubitzer (2013) and Krubitzer (2009)]. Colored areas in brain illustrations indicate primary sensory (red: somatosensory; blue: visual; yellow: auditory) areas in representative extant primate and rodent species of body (first numbers in brackets) and brain (last numbers in brackets) sizes. Note the difference in proportion of these primary areas and association areas (in white) in different sized brains between primates and rodents

The SII resides along the lateral sulcus in the opercular tegmentum, one of the first and most expanded primate brain areas during evolution of human ancestors. Primates’ cortical expansion was achieved through an increase in numbers of neurons and resulting additions of redundant but new cortical areas, in contrast to rodents (the order closest to primates among mammals), in which larger brains are structurally analogous to smaller ones (Herculano-Houzel 2012). Thus, human SII specificity could be speculated as a direct extrapolation of non-human primate SII, yet substantially expanded to bridge the gap between human and non-human SII functions (Fig. 3).

### Genetic characteristics of the primate cortex: a site for high adaptability

Primate brains, having unique design principles (such as genetic expression patterns across cortical areas, particularly during early development), unsurprisingly allow more plastic adaptability over rodents. In situ hybridization of cadherin family molecules (cell adhesion molecules involved in specific synapse formation and function) was compared between developing marmosets and mice (Matsunaga et al. 2013). In mice, type-II cadherins showed broad and overlapping expressions within each other, starting from the embryonic stage and continuing during postnatal development. In marmosets, in contrast, broad and overlapping cadherin expressions soon became localized to restricted areas of the brain, showing distinct expression levels among each other during development, suggesting refinement or functional differentiation of cortical areas. Thus, restricted/less redundant cadherin expression might allow the primate brain functional diversity, accounting for the differences observed in comparison to the rodent brain. Furthermore, specific to primates during the embryonic stage, each cadherin showed a distinct spatial and temporal expression. These results suggest that the differential expression of diverse cadherins is involved in primate-specific cortical development (Fig. 7 in Matsunaga et al. 2015b).

Another factor that affects plasticity during postnatal development is periostin (osteoblast specific factor), which was recently reported to function in axon regeneration and neuroprotection. Through a comparative analysis, periostin was expressed at higher rates in primates than in mice, in which overexpression induced neurite outgrowth. The results suggest that prolonged and increased periostin expression in the primate cerebral cortex enhances cortical plasticity beyond the typically observed in mammals (Matsunaga et al. 2015c). Moreover, postnatal plasticity is not only regulated by genomic information, but also through epigenetic modification as an activity-dependent mechanism. Active DNA methylation/demethylation of promoter regions is one of the epigenetic mechanisms that can modify gene expression, in which Gadd45 alpha, beta, and gamma have been identified, among others, as regulators. In the marmoset, the cortical expression of Gadd45 alpha and gamma is reduced during development, whereas increased expression of Gadd45 has been observed in some areas of adults, including prefrontal, temporal, posterior parietal, and insular cortices, which are particularly expanded in greater primates and humans. Compared to in the marmoset brain, no clear regional differences and constant or reduced Gadd45 expression was observed in both juvenile and adult mouse brains (Matsunaga et al. 2015a).

### Body axis of primates: vertical conversion required higher spatial integration

The primate cortex flexibility may have arisen from the need to adapt to an arboreal habitat, which for the first time required sophisticated visuo-motor coordination with accurate depth analyses of three-dimensional (3D) space. Indeed, the middle temporal visual area (MT/V5) is particularly evolved in the primate brain, which is crucial for 3D motion processing. As an extension to the studies depicted in the previous section, shifts in cadherin expression patterns were detected between pulvino-MT and lateral geniculate nucleus (LGN)-V1 pathways and in the MT/V5 around the time of birth, which preceded the development of V1-MT connections, suggesting that a change in cadherin expression around the lateral sulcus may be a general mechanism to control neural plasticity in regard to higher cognitive abilities (Fig. 5 in Matsunaga et al. 2014).

Another rather notable primate behavior is the “sitting position”, allowing the dexterous usage of hands as apparatuses to reach and grasp, diverging from their original locomotor function. This behavior raised the body axis vertically, preceding bipedal locomotion, and dramatically increased the diversity and orientations of reach-and-grasp motions in the space around the body axis. However, it required complex information processing by the brain to harmonize the movement of different body parts by translating positional information between different coordinate systems (Iriki and Taoka 2012). In this way, one can speculate the rationale of opercular expansion (within which resides the SII) to integrate multiple modalities of information for conceptual transformation of the relationships between the self and the environment. Altogether, parietal integration represents structures of the body itself through the organized composition of multiple body parts and their flexible and voluntary modifications, whereas SII represents the relationship of the self-body as a whole in relation to the holistic environmental structures under abstract spatial dimensions (Corradi-Dell’Acqua et al. 2009). Thus, as expected, primate SII was found to expand by tool-use training, which requires conscious mastery of one's own image in a sitting position (Quallo et al. 2009).

### Primates’ reuse of mammalian SII: somatocentric information integration

Evolution proceeds through two different paths: (1) species with short life spans and mass reproduction adapt to the environment through variations in their numerous offspring, expecting at least a few to survive, whereas (2) species with long life spans and low birth rates adapt to the environment through an individual capacity to adapt, which is carried out by the expansion of an organ for adaptive behaviors. The primate brain, and that of humans in particular, is the representative example of the latter. The primate brain was enlarged by increased numbers of neurons and thereby additions of novel cortical areas, which diverged from existing ones resulting in increased inter-areal connections (Herculano-Houzel 2012), eventually creating novel functions. A slightly excessive redundancy of the brain to stabilize a system against unexpected environmental noise occasionally allows the system to be bistable between the original and newly acquired state enabling networks to be used for different functions, maybe in combination with other parts of the brain (Iriki 2010). Such mechanisms have been proposed as the theory of neural reuse or recycling (Dehaene and Cohen 2007; Anderson 2010), which claims that neural circuits established for one purpose are exapted (exploited, recycled, redeployed) during evolution or normal development, being put to different uses, often without losing their original functions. The SII of human and non-human primates under this perspective would be an exemplar representation of such process. Human specific SII functions (“Somatotopic functions of SII and adjacent opercular cortex: a conventional view”) are specialized for cultural domains that do not exist in non-human primates. The proposed neural reuse/recycling hypothesis may have permeated the evolutionarily older brain circuits of SII to accomplish this task while inheriting many of their structural constraints, being yet essentially a center for higher-order somatosensory information processing.

## Expansion of the information space: transition from body map to body-in-the-world map

Then, what could be the governing principle of information processing in the primate SII, and its functional significance, if anything beyond a straightforward physical body map, to bridge the current gaps between the human and non-human brains?

It seems that, although at a complex level, SII is essentially closer to the somatosensory input side of the cerebral cortex (via strong cortico-cortical connections) than to the motor output side, unlike in the posterior parietal cortex where response properties resemble SII (“Multiple body maps”, “Sensorimotor integration for object manipulation and active tactile discrimination”, “Multimodal integrations”). This results in, applying the principles found in SI onto SII neuronal function (e.g., modes of neuronal receptive field properties), vague and ambiguous somatotopic representations of the physical body map, suggesting that this information is only loosely connected to bodily structures (“Multiple body maps”, “Multimodal integrations”, “Whole body somatic sensory integration”). This indicates that SII function not only involves conventional body mapping, but that it has other additional, previously unknown/unrecognized functions somewhat related to it, with more research being necessary to uncover the range of such functions in the non-human primate brain.

The additional elements that shape SII activity should integrate with other sensory modalities representing relationships between the body in itself and the outside world, including its spatial structures. Dissimilar dimensions, as well as their respective temporal aspects, such as vestibular, visceral, and visual information, establish associations between the inner and outer worlds (“Multisensory integration”, “Multimodal integrations”). The highly plastic and dynamic nature of primate SII neuronal circuitry seems to allow the incorporation of newly acquired functions as an extension of existing ones during the development, learning, and evolutionary processes that interconnect various aspects of the self-body state into extra-body information (“Body schema and tool use induced morphological plasticity”, “Genetic characteristics of the primate cortex: a site for high adaptability”, “Body axis of primates: vertical conversion required higher spatial integration”).

Then, what induced this drastic, phase transition-like, discontinuous expansion in human lineage? Its latent basis must have been already present in the primate brain, as its mode of expansion shifted to allow additional new brain areas to emerge upon expansion (Fig. 3). A diversion in this expansion rate between human and non-human primates (orange line) and its following surge seem to have initiated when ancestral hominids started using and manufacturing stone tools (Fig. 3, red line and oblique arrow). Through the active mechanism (in contrast to the passive, underlying natural selection) of *triadic niche construction*, an interactively accelerated feedback loop for the expansion of neural, cognitive and environmental niches occurred (Iriki and Taoka 2012). SII must be among the main brain areas expanded by this interaction (Quallo et al. 2009), as explained in “Body schema and tool use induced morphological plasticity”. In this way, human evolution was characterized by a continuous process of addition of new categories of cognitive capacity, including those related to the manufacture and usage of tools and the establishment of linguistic faculties, supported by the dramatic brain expansion that accompanied the addition of new functional areas surrounding the existing ones. Such extended brain functions have driven rapid and drastic changes in the hominin ecological niche, which in turn demanded further brain resources to adapt to it. Increased relations between body parts and the tools, initially objects in the external world, were established, precipitating mental mechanisms of self-objectification (Iriki 2006), forming the precursory neural bases of self-consciousness, which is recognizing oneself as an existence in the outside world.

Thus, the non-somatotopic information processing of human SII appears to be a direct extrapolation and reuse/recycling of the above-advanced somatic sensory processing by applying the same principle of representing intra-bodily schemes to include the extra-bodily space (in its physical and social environments) in a somatocentric manner, possibly under the conscious perception of both the self-body and the environment (“Tactile perception”, “Higher-order functions”, “Body schema and tool use induced morphological plasticity”, “Primates’ reuse of mammalian SII—somatocentric information integration”)—namely as a body-in-the-world map. With this map, humans are able to explicitly describe and allocate their bodily states (including posture, location, conditions, emotions, perceptions, and feelings) in relation to the external world, and its reflection projects those internal states onto the outside world as a function of the projection process (“The notion of projection”) depicted at the beginning of this article to create the conscious experience of the self-in-the-world.

## Conclusion: conscious experience of the self in the world

This article summarized current gaps in knowledge that contrast the human and non-human primates' SII function, overviewing recent neuroscientific evidence in relation to possible evolutionary aspects. As a result, the supposedly unique characteristics of the human SII could be explained as a continuation of primate brain development, bridging this functional gap. Taken this information, we would like to propose that a primate’s SII (perhaps including the adjacent parietal opercular cortex) is a site of information integration, grounded essentially in spatial body maps, yet allowing humans and other primates to also form complex relations between the body and the environmental space. This ability, supported by this region's genetically acquired flexibility to incorporate related information, would allow expanding spatial dimensions over the existing body map, establishing the body-in-the-world map. More importantly, the relativism of the body in relation to the surrounding environment might comprise the neural basis for consciousness, situating the self in the world and those together as a whole, while also serving as the observer of the SI homunculus (precise somatotopic map), avoiding an infinite regression, as the brain’s internal processes establishes this homunculus. Such a phase transition seems to have been triggered by a brain expansion surge in ancestral hominids coinciding with the onset of tool use, which establishes a relationship of equivalence between the body parts and the tools (external objects), whereby mental mechanisms of self-objectification might have emerged.

The map represented in SII is not merely a physically straightforward one, but rather it is also furnished with varieties of complex concrete and abstract concepts found in nature (including multimodality, flexibility, polysemantics, and arbitrariness), consciously choosing and creating relationships (a function of projection) as the immediate conditions the self faces in the demands of the external world. In this sense, the self shapes the environment and the reach of these modifications are incorporated as part of the self, integrating corporeal, environmental and cognitive limits. As such, we would like to propose the concept of a somatocentric holistic self, represented as a body-in-the-world map in the primate SII. This concept blurs the border between body and environment by assuming the world as perceived is a product of the self. These mechanisms might comprise the neural underpinnings of the emergence of self-consciousness, by which most of the recently found human SII functions, which initially seemed not directly connected to non-human SII empirical studies, can be reasonably explained.

## References

[CR1] Alais D, Burr D (2004). The ventriloquist effect results from near-optimal bimodal integration. Curr Biol.

[CR2] Anderson ML (2010). Neural reuse: a fundamental organizational principle of the brain. Behav Brain Sci.

[CR3] Bailenson J (2018). Experience on demand: what virtual reality is, how it works, and what it can do.

[CR101] Baier B, Karnath HO (2008). Tight link between our sense of limb ownership and self-awareness of actions. Stroke.

[CR5] Binkofski F, Buccino G, Stephan KM, Rizzolatti G, Seitz RJ, Freund H-J (1999). A parieto-premotor network for object manipulation: evidence from neuroimaging. Exp Brain Res.

[CR6] Blakemore SJ, Bristow D, Bird G, Frith C, Ward J (2005). Somatosensory activations during the observation of touch and a case of vision–touch synaesthesia. Brain.

[CR7] Bottini G, Gandola M (2015). Beyond the non-specific attentional effect of caloric vestibular stimulation: evidence from healthy subjects and patients. Multisens Res.

[CR8] Bottini G, Paulesu E, Sterzi R, Warburton E, Wise RJS, Vallar G, Frackowiak RSJ, Frith CD (1995). Modulation of conscious experience by peripheral sensory stimuli. Nature.

[CR9] Botvinick M, Cohen J (1998). Rubber hands ‘feel’ touch that eyes see. Nature.

[CR10] Brozzoli C, Gentile G, Ehrsson HH (2012). That’s near my hand! Parietal and premotor coding of hand-centered space contributes to localization and self-attribution of the hand. J Neurosci.

[CR11] Burton H, Fabri M, Alloway K (1995). Cortical areas within the lateral sulcus connected to cutaneous representations in areas 3b and 1: a revised interpretation of the second somatosensory area in macaque monkeys. J Comp Neurol.

[CR12] Cipolloni PB, Pandya DN (1999). Cortical connections of the frontoparietal opercular areas in the rhesus monkey. J Comp Neurol.

[CR13] Corradi-Dell’Acqua C, Tomasino B, Fink GR (2009). What is the position of an arm relative to the body? Neural correlates of body schema and body structural description. J Neurosci.

[CR14] Dehaene S, Cohen L (2007). Cultural recycling of cortical maps. Neuron.

[CR15] Devue C, Collette F, Balteau E, Degueldre C, Luxen A, Maquet P, Brédart S (2007). Here I am: the cortical correlates of visual self-recognition. Brain Res.

[CR16] Disbrow E, Litinas E, Recanzone GH, Padberg J, Krubitzer L (2003). Cortical connections of the second somatosensory area and the parietal ventral area in macaque monkeys. J Comp Neurol.

[CR17] Dooley JC, Krubitzer L (2013). Cortical plasticity within and across lifetimes: how can development inform us about phenotypic transformations?. Front Hum Neurosci.

[CR18] Eickhoff SB, Schleicher A, Zilles K, Amunts K (2006). The human parietal operculum. I. Cytoarchitectonic mapping of subdivisions. Cereb Cortex.

[CR19] Eickhoff SB, Jbabdi S, Caspers S, Laird AR, Fox PT, Zilles K, Behrens TEJ (2010). Anatomical and functional connectivity of cytoarchitectonic areas within the human parietal operculum. J Neurosci.

[CR20] Evolution du volume cérébral des Hominidés (2016) https://www.linternaute.com/science/biologie/dossiers/06/0608-memoire/8.shtml. Accessed 29 Jan 2019

[CR21] Ferrè ER, Haggard P (2015). Vestibular–somatosensory interactions: a mechanism in search of a function?. Multisens Res.

[CR22] Ferrè ER, Bottini G, Haggard P (2012). Vestibular inputs modulate somatosensory cortical processing. Brain Struct Funct.

[CR23] Fitzgerald PJ, Lane JW, Thakur PH, Hsiao SS (2004). Receptive field properties of the macaque second somatosensory cortex: evidence for multiple functional representations. J Neurosci.

[CR24] Frith C, Lawrence A, Weinberger D (1996). The role of the prefrontal cortex in self-consciousness: the case of auditory hallucinations [and discussion]. Philos Trans Biol Sci.

[CR25] Geiger J (2009). The third man factor: the secret to survival in extreme environments.

[CR26] Guipponi O, Cléry J, Odouard S, Wardak C, Ben Hamed S (2015). Whole brain mapping of visual and tactile convergence in the macaque monkey. NeuroImage.

[CR27] Hafting T, Fyhn M, Molden S, Moser M-B, Moser EI (2005). microstructure of a spatial map in the entorhinal cortex. Nature.

[CR28] Hashimoto T, Iriki A (2013). Dissociations between the horizontal and dorsoventral axes in body-size perception. Eur J Neurosci.

[CR29] Herculano-Houzel S (2012). The remarkable, yet not extraordinary, human brain as a scaled-up primate brain and its associated cost. Proc Natl Acad Sci.

[CR30] Hihara S, Taoka M, Tanaka M, Iriki A (2015). Visual responsiveness of neurons in the secondary somatosensory area and its surrounding parietal operculum regions in awake macaque monkeys. Cereb Cortex.

[CR31] Hinkley LB, Krubitzer LA, Nagarajan SS, Disbrow EA (2007). Sensorimotor integration in S2, PV, and parietal rostroventral areas of the human sylvian fissure. J Neurophysiol.

[CR32] Hogendoorn H, Kammers M, Haggard P, Verstraten F (2015). Self-touch modulates the somatosensory evoked P100. Exp Brain Res.

[CR33] Holle H, Banissy MJ, Ward J (2013). Functional and structural brain differences associated with mirror-touch synaesthesia. NeuroImage.

[CR34] Hsiao S (2008). Central mechanisms of tactile shape perception. Curr Opin Neurobiol Sens Syst.

[CR35] Ionta S, Heydrich L, Lenggenhager B, Mouthon M, Fornari E, Chapuis D, Gassert R, Blanke O (2011). Multisensory mechanisms in temporo-parietal cortex support self-location and first-person perspective. Neuron.

[CR36] Iriki A (2006). The neural origins and implications of imitation, mirror neurons and tool use. Curr Opin Neurobiol Motor Syst Neurobiol Behav.

[CR37] Iriki A (2010). Neural reuse: a polysemous and redundant biological system subserving niche-construction. Behav Brain Sci.

[CR38] Iriki A, Taoka M (2012). Triadic (ecological, neural, cognitive) niche construction: a scenario of human brain evolution extrapolating tool use and language from the control of reaching actions. Philos Trans R Soc B.

[CR100] Ishida H, Nakajima K, Inase M, Murata A (2010). Shared mapping of own and others' bodies in visuotactile bimodal area of monkey parietal cortex. J Cognit Neurosci.

[CR39] Karnath H-O, Baier B, Nägele T (2005). Awareness of the functioning of one’s own limbs mediated by the insular cortex?. J Neurosci.

[CR40] Keysers C, Kaas JH, Gazzola V (2010). Somatosensation in social perception. Nat Rev Neurosci.

[CR41] Krubitzer L (2009). In search of a unifying theory of complex brain evolution. Ann N Y Acad Sci.

[CR42] Krubitzer L, Clarey J, Tweedale R, Elston G, Calford M (1995). A redefinition of somatosensory areas in the lateral sulcus of macaque monkeys. J Neurosci.

[CR43] Lacey S, Stilla R, Sathian K (2012). Metaphorically feeling: comprehending textural metaphors activates somatosensory cortex. Brain Lang.

[CR44] Leigh R, Oishi K, Hsu J (2013). Acute lesions that impair affective empathy. Brain.

[CR45] Lopez C, Halje P, Blanke O (2008). Body ownership and embodiment: Vestibular and multisensory mechanisms. Neurophysiol Clin Clin Neurophysiol.

[CR46] Lopez C, Schreyer H-M, Preuss N, Mast FW (2012). Vestibular stimulation modifies the body schema. Neuropsychologia.

[CR47] Matsunaga E, Nambu S, Oka M, Okanoya K, Iriki A (2013). Comparative analysis of protocadherin-11 X-linked expression among postnatal rodents, non-human primates, and songbirds suggests its possible involvement in brain evolution. PLoS ONE.

[CR48] Matsunaga E, Nambu S, Oka M, Iriki A (2014). Complementary and dynamic type II cadherin expression associated with development of the primate visual system. Dev Growth Differ.

[CR49] Matsunaga E, Nambu S, Oka M, Iriki A (2015). Comparative analysis of developmentally regulated expressions of Gadd45a, Gadd45b, and Gadd45g in the mouse and marmoset cerebral cortex. Neuroscience.

[CR50] Matsunaga E, Nambu S, Oka M, Iriki A (2015). Complex and dynamic expression of cadherins in the embryonic marmoset cerebral cortex. Dev Growth Differ.

[CR51] Matsunaga E, Nambu S, Oka M, Tanaka M, Taoka M, Iriki A (2015). Periostin, a neurite outgrowth-promoting factor, is expressed at high levels in the primate cerebral cortex. Dev Growth Differ.

[CR52] Matsunaga E, Nambu S, Oka M, Tanaka M, Taoka M, Iriki A (2015). Identification of tool use acquisition-associated genes in the primate neocortex. Dev Growth Differ.

[CR53] Maule F, Barchiesi G, Brochier T, Cattaneo L (2015). Haptic working memory for grasping: the role of the parietal operculum. Cereb Cortex.

[CR54] Nummenmaa L, Smirnov D, Lahnakoski JM, Glerean E, Jääskeläinen IP, Sams M, Hari R (2014). Mental action simulation synchronizes action-observation circuits across individuals. J Neurosci.

[CR55] O’Keefe J, Nadel L (1978). The hippocampus as a cognitive map.

[CR56] Polanyi M (1967). The tacit dimension.

[CR57] Preusser S, Thiel SD, Rook C, Roggenhofer E, Kosatschek A, Draganski B, Blankenburg F, Driver J, Villringer A, Pleger B (2015). The perception of touch and the ventral somatosensory pathway. Brain.

[CR58] Pylyshyn ZW (2011). Things and places: how the mind connects with the world.

[CR59] Quallo MM, Price CJ, Ueno K, Asamizuya T, Cheng K, Lemon RN, Iriki A (2009). Gray And White Matter Changes Associated With Tool-Use Learning In Macaque Monkeys. Proc Natl Acad Sci.

[CR60] Raos V, Kilintari M, Savaki HE (2014). Viewing a forelimb induces widespread cortical activations. NeuroImage.

[CR61] Robinson CJ, Burton H (1980). Somatotopographic organization in the second somatosensory area of *M. Fascicularis*. J Comp Neurol.

[CR62] Romo R, de Lafuente V (2013). Conversion of sensory signals into perceptual decisions. Prog Neurobiol Conv Sens Signals Percept Mem Decis.

[CR63] Singer T, Seymour B, O’Doherty J (2004). Empathy for pain involves the affective but not sensory components of pain. Science.

[CR64] Taoka M, Toda T (2004) Cortico-cortical connections of the hand region in the second somatosensory cortex of the japanese macaque monkey. Neuroscience meeting planner. Program No. 59.12. San Diego, CA

[CR65] Taoka M, Tanaka M, Hihara S, Ojima H, Iriki A (2013). Neural response to movement of the hand and mouth in the secondary somatosensory cortex of Japanese monkeys during a simple feeding task. Somatosens Mot Res.

[CR66] Taoka M, Toda T, Hihara S, Tanaka M, Iriki A, Iwamura Y (2016). A Systematic analysis of neurons with large somatosensory receptive fields covering multiple body regions in the secondary somatosensory area of macaque monkeys. J Neurophysiol.

[CR67] Tsakiris M, Hesse MD, Boy C, Haggard P, Fink GR (2007). Neural signatures of body ownership: a sensory network for bodily self-consciousness. Cereb Cortex.

[CR68] Valenza N, Ptak R, Zimine I, Badan M, Lazeyras F, Schnider A (2001). Dissociated active and passive tactile shape recognition: a case study of pure tactile apraxia. Brain.

